# Machine Learning for Nuclear Mechano-Morphometric Biomarkers in Cancer Diagnosis

**DOI:** 10.1038/s41598-017-17858-1

**Published:** 2017-12-20

**Authors:** Adityanarayanan Radhakrishnan, Karthik Damodaran, Ali C. Soylemezoglu, Caroline Uhler, G. V. Shivashankar

**Affiliations:** 1 Department of Electrical Engineering and Computer Science, Laboratory for Information and Decision Systems, Institute for Data, Systems and Society, MIT, Cambridge, MA USA; 2 Mechanobiology Institute and Department of Biological Sciences, National University of Singapore, Singapore, Singapore; 3 FIRC Institute for Molecular Oncology (IFOM), Milan, Italy

## Abstract

Current cancer diagnosis employs various nuclear morphometric measures. While these have allowed accurate late-stage prognosis, early diagnosis is still a major challenge. Recent evidence highlights the importance of alterations in mechanical properties of single cells and their nuclei as critical drivers for the onset of cancer. We here present a method to detect subtle changes in nuclear morphometrics at single-cell resolution by combining fluorescence imaging and deep learning. This assay includes a convolutional neural net pipeline and allows us to discriminate between normal and human breast cancer cell lines (fibrocystic and metastatic states) as well as normal and cancer cells in tissue slices with high accuracy. Further, we establish the sensitivity of our pipeline by detecting subtle alterations in normal cells when subjected to small mechano-chemical perturbations that mimic tumor microenvironments. In addition, our assay provides interpretable features that could aid pathological inspections. This pipeline opens new avenues for early disease diagnostics and drug discovery.

## Introduction

Recent studies have revealed that cells within the tissue microenvironment are subjected to a variety of mechanical and chemical signals that regulate cellular homeostasis^1–3^. The cell nucleus operates as an integrator of these signals via an elaborate meshwork of cytoskeletal-to-nuclear links^4–6^. In normal cells, these links maintain the nuclear mechanical homeostasis to regulate genomic programs. Alterations in mechano-chemical signals to the cell nucleus have been shown to play a critical role for the onset of various diseases^7–11^. Importantly, cancer cells exhibit structural, mechanical, and conformational aberrations in their nuclear architecture, including altered nuclear envelope organization^12–14^, distinct chromatin arrangements^15,16^, and telomere stability^17^. Cancer tissues have been conjectured to arise from single cells within the tissue microenvironment, in which impaired nuclear mechanotransduction resulted in altered genomic programs^18–20^.

Nuclear morphometric information is one of the major clinical diagnostic approaches used by pathologists to determine the malignant potential of cancer cells^21–25^. However, since the tissue microenvironment is highly heterogeneous, such morphometric assays are currently subjective and depend on the interpretation of the pathologist^26^. To circumvent this subjectivity, recent advances in imaging combined with machine learning have provided new avenues^27–32^. Given the prominent role of nuclear structure changes in cancer cells, various machine learning techniques based on quantitative nuclear morphometric information such as nuclear size, shape, nucleus-to-cytoplasm ratio, and chromatin texture, and various non-parametric methods such as deep learning have been applied for classifying histopathology images and diagnosing various cancers including breast, skin and thyroid cancer^33–41^. While this line of research has allowed accurate late-stage prognosis, early diagnosis remains a major challenge. This is primarily due to the difficulty of detecting a small number of abnormal cells in a heterogeneous population of normal cells as this is the case for example in fine-needle biopsies or blood smears. In addition, while there are accurate deep learning approaches, they don’t provide any interpretable features and functional annotations that could be used by pathologists for early disease diagnosis.

In this paper, we first present a pipeline combining single-cell imaging with machine learning algorithms to discriminate between normal and cancer cell lines. In particular, we develop a convolutional neural net pipeline that not only provides high classification accuracy but also interpretable features. This is achieved by analyzing small regions within the nucleus using our deep learning pipeline and correlating these with changes in heterochromatin to euchromatin ratios in normal and cancer cell lines. In addition, we validate our pipeline by discriminating between normal and cancer tissue slices. Importantly, we also evaluate the sensitivity of our method on normal cells subjected to small mechanical and biochemical perturbations to mimic the chromatin condensation patterns in early stages of cancer. Collectively, our platform has potential applications for drug discovery and early disease diagnostics in human populations.

## Results

### SCENMED: A combined fluorescence imaging and convolutional neural net platform for classifying nucleus images

**Figure 1:**
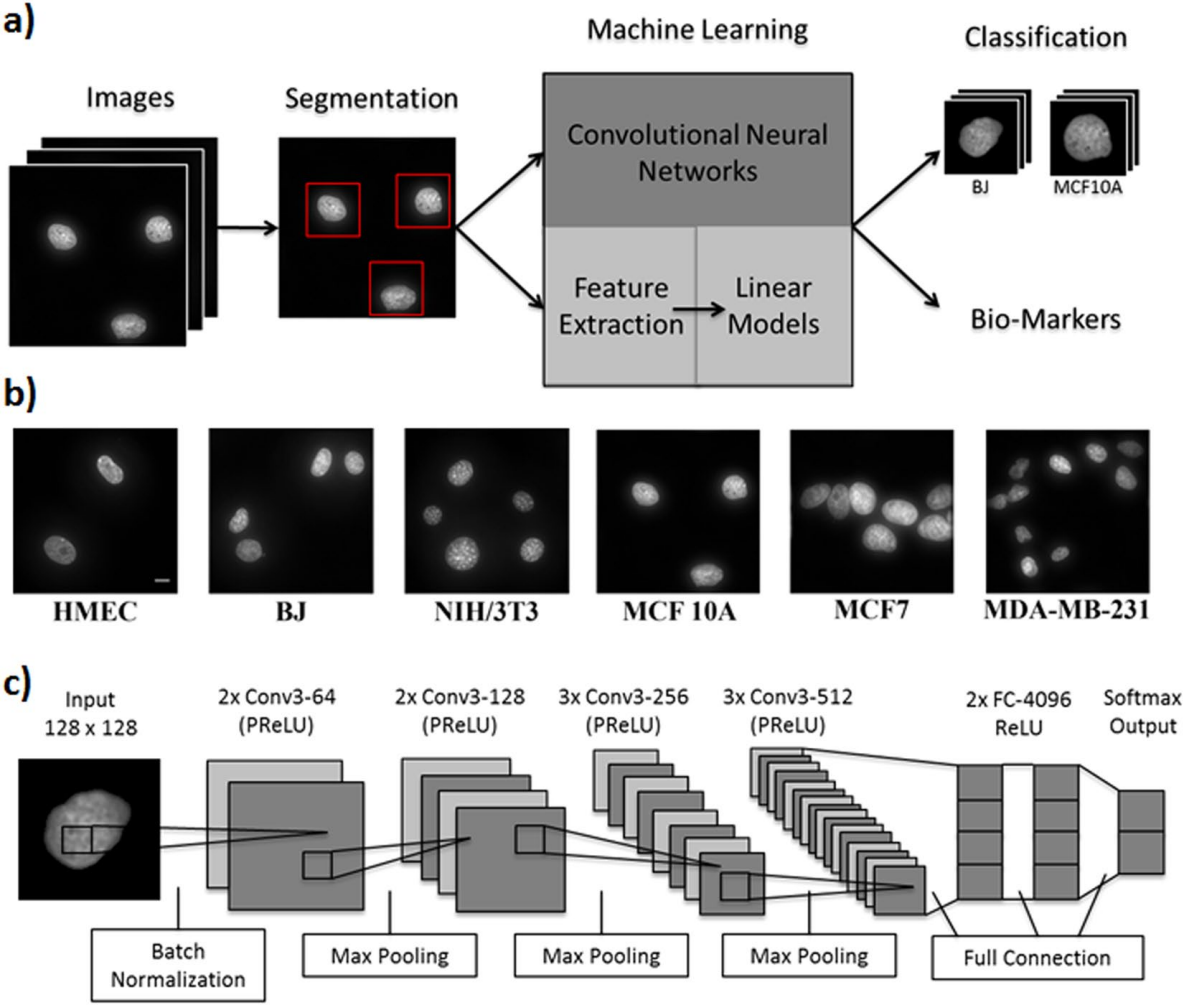
Single-cell nuclear mechanical diagnostics (SCENMED) platform for cancer prognosis. **(a)** Analysis pipeline to identify nuclei and extract nuclear morphometric features. Cells are first fixed and imaged. The images are then classified using a linear model based on the extracted nuclear morphometric features and a convolutional neural network based on the segmented images. **(b)** Representative images of nuclei for NIH/3T3, BJ, HMEC, MCF10A, MCF7, and MDA-MB-231. **(c)** Adaptation of the VGG Convolutional Neural Network architecture consisting of an initial batch normalization layer followed by 10 layers of convolutions, 4 max pooling layers (kernel size 2, stride size 2), 2 fully connected layers, and a softmax output layer. Each convolution is followed by PReLU activations, and the fully connected layers are followed by ReLU activations.

We developed a single-cell nuclear mechanical diagnostics (SCENMED) platform consisting of single-cell fluorescence imaging and machine learning to discriminate between normal and cancer cells. Our pipeline is shown in Fig. 1a. First, cells were seeded in glass bottom dishes and were allowed to grow overnight. The cells were then fixed, permeabilized, and stained with DAPI (4′,6-diamidino-2-phenylindole). Several thousand wide-field images of the nucleus were acquired using a 60X objective. Representative nucleus images of the different cell types (NIH/3T3, BJ, MCF 10A, MCF7, MDA-MB-231, HMEC) are shown in Fig. 1b. The individual nuclei were identified using a 4-step image processing procedure to remove overexposure, edge blur and drift blur (see Methods and Supplementary Fig. 1a and Fig. 1b). These images were then classified using an approach based on an adaptation of the VGG (Visual Geometry Group) Convolutional Neural Network architecture (see Fig. 1c) and a supervised approach using linear models based on morphometric features that were extracted from each nucleus. These morphometric features capture shape and texture of the nucleus (see Methods) and have also been used in previous cancer classification studies^21,30^.

### SCENMED discriminates between normal and cancer cell lines with high accuracy

**Figure 2:**
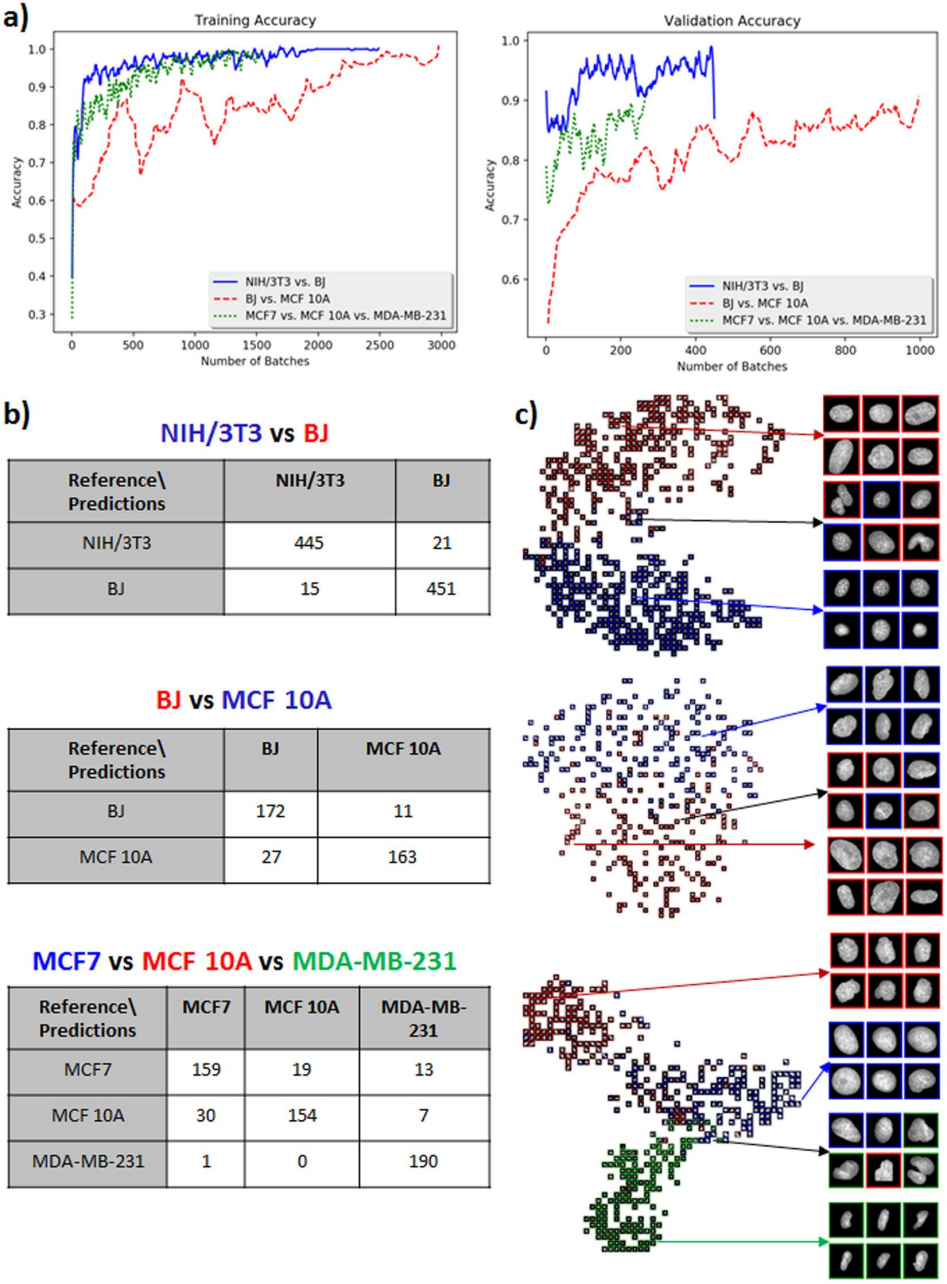
Classification of NIH/3T3 versus BJ, BJ versus MCF10A, and MCF10A versus MCF7 versus MDA-MB-231 nuclei. **(a)** Training and validation accuracies of the neural model for the classification tasks of NIH/3T3 versus BJ, BJ versus MCF10A, and MCF7 versus MCF 10A versus MDA-MB-231 nuclei. **(b)** Confusion matrices for the three different classification tasks. **(c)** t-SNE visualizations for the three different classification tasks.

As a first step, we used our pipeline to discriminate between normal mouse and normal human fibroblast cells (NIH/3T3 and BJ) and various human breast cancer cell lines (MCF10A, MCF7 and MDA-MB-231). These representative breast cancer cell lines comprise of fibrocystic as well as metastatic states. The number of cell nuclei imaged in each condition and the split into training and validation is given in Supplementary Fig. 3a. As shown in Fig. 2a, validation accuracies for the neural model revealed that mouse versus human cell lines and normal versus cancer cell lines can be robustly discriminated with a large margin (NIH/3T3 versus BJ: 96.1% validation accuracy and 97.6% training accuracy; BJ versus MCF10A: 88.2% validation accuracy and 93.8% training accuracy). As expected, the progression of the training accuracy over the different batches shows that the neural model learns more quickly to distinguish between NIH/3T3 and BJ cells as compared to BJ versus MCF10A cells. In addition, Fig. 2a also shows that the three breast cancer cell lines can be discriminated with high accuracy (MCF7 versus MCF10A versus MDA-MB-231: 87.8% validation accuracy and 96.2% training accuracy. Counter intuitively, validation accuracy and validation loss might both increase, since the model is simply oscillating between class predictions for the cases that are difficult to distinguish (see also Supplementary Fig. 3b). A confusion matrix can be used to further quantify the discriminative power of classification methods and is shown in Fig. 2b. As expected, the most aggressive cancer cell line (MDA-MB-231) is easy to distinguish from the fibrocystic (MCF10A) and less aggressive cancer cell line (MCF7). Further, we used t-distributed stochastic neighbor embedding (t-SNE) to embed the high-dimensional data resulting from the neural net model into two dimensions and visualize the classification between the various cell lines. In these t-SNE plots similar cell nuclei are represented by nearby points, whereas dissimilar cell nuclei correspond to distant points (Fig. 2c). As can be clearly seen in the confusion matrices and the t-SNE plots, our method can easily distinguish between fibroblasts of mouse and human origin, human fibroblast and cancer cells, as well as the three cancer cell lines.

### SCENMED platform applied to patches provides interpretable features for cancer diagnosis

The importance of different morphometric features for each classification task was determined using a regularized logistic regression analysis and is displayed in Table 1. Our analysis of various texture and shape features shows that texture features were most discriminative for classifying mouse versus human fibroblasts and for the different cancer cell lines, whereas shape features were most discriminative for classifying BJ versus MCF10A nuclei. As to be expected, the regularized logistic regression approach yields slightly lower discriminative power as compared to the convolutional neural net approach. However, it has the advantage that it provides a list of interpretable features together with their importance for classification.

**Table 1:** Feature ablation tables based on a linear model for all three classification tasks indicating the effect on training and validation accuracy when removing each feature (or set of features).

NIH/3T3 vs. BJ		BJ vs. MCF 10 A		MCF7 vs. MCF 10 A vs. MDA-MB-231	
Features Used	Training/ Validation Accuracy	Features Used	Training, Validation Accuracy	Features Used	Training/Validation Accuracy
Features Used	Training/ Validation Accuracy	Features Used	Training, Validation Accuracy	Features Used	Training/Validation Accuracy
All features	96.4%,95.1%	All features	89.1%, 83.9%	All features	93.7%, 89.0%
- Texture features	81.5%, 76.5%	- Shape features	72.9%, 61.1%	- Texture features	80.0%, 69.8%
- Shape features	95.7%, 94.9%	- Texture features	87.8%, 82.1%	- Shape features	81.1%, 79.8%
All features	96.4%, 95.1%	All features	89.1%, 83.9%	All features	93.7%, 89.0%
- PFTAS	93.1%, 90.6%	- PFTAS	85.7%, 81.3%	- area	80.7%, 79.2%
- LBP	95.6%, 93.3%	- LBP	85.5%, 82.4%	- PFTAS	89.4%, 82.7%
- roundness	96.3%, 94.8%	- area	88.9%, 82.9%	- LBP	92.2%, 86.6%
-Major axis length	96.4%, 94.8%	- eccentricity	88.9%, 83.2%	- Zernike Moments	92.2%, 87.1%
-eccentricity	96.4%, 94.8%	- roundness	88.8%, 83.9%	- Minor axis length	93.4%, 88.8%
- Minor axis length	96.4%, 95.0%	- Major axis length	88.9%, 83.9%	- Major axis length	93.5%, 88.5%
- Zernike Moments	95.5%, 95.1%	- Minor axis length	88.9%, 83.9%	- roundness	93.7%, 88.5%
- area	96.4%, 95.1%	- Zernike Moments	88.7%, 84.2%	- eccentricity	93.5%, 89.0%

**Figure 3:**
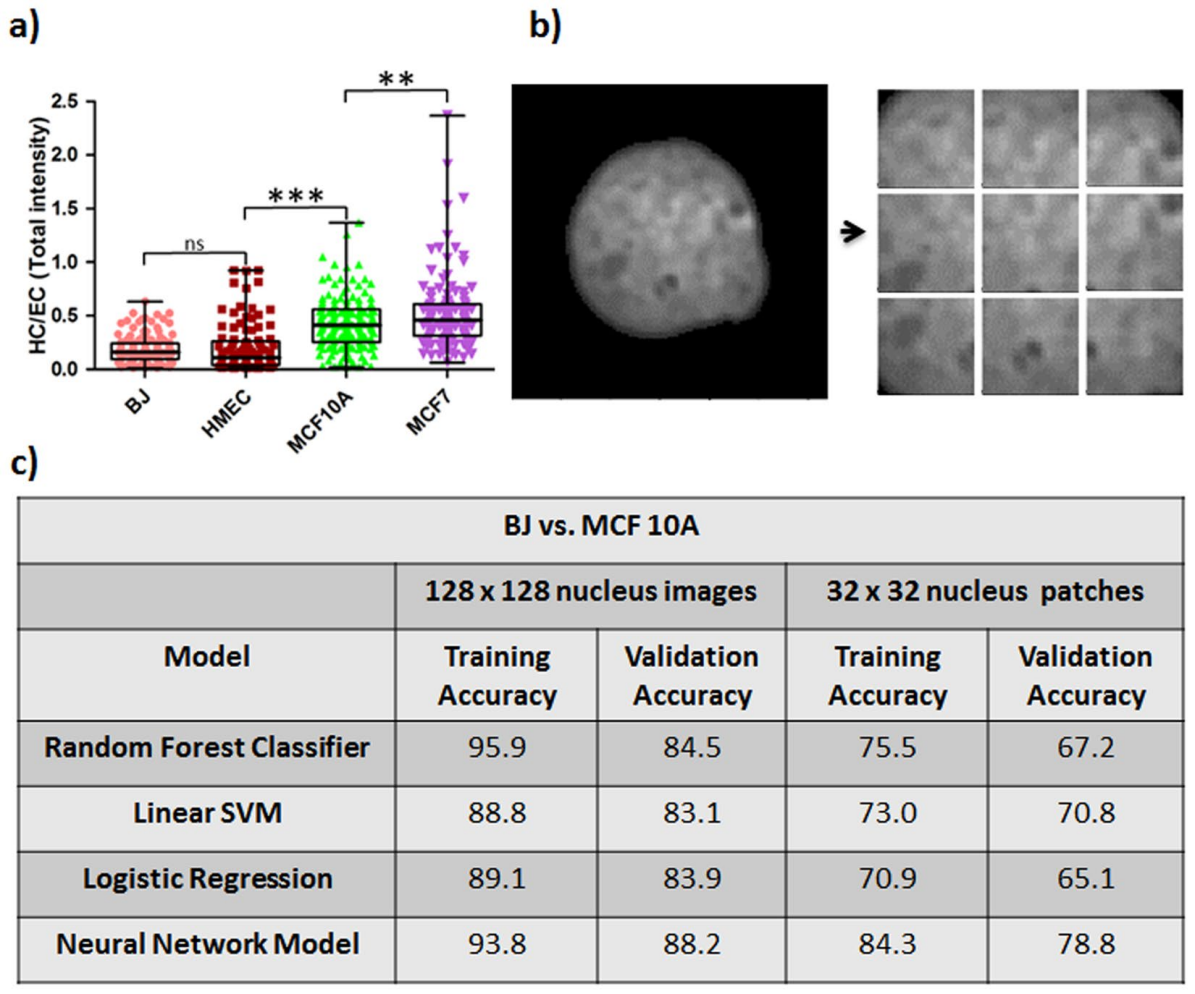
Identification of informative nuclear morphometric features for cancer prognosis. **(a)** Whisker box plot for HC/EC of normal and cancer cell lines (BJ: n = 134, HMEC: n = 101, MCF10A: n = 156, MCF7: n = 112; Student’s unpaired t-test ***p < 0.0001, **p < 0.0001 and ns = not significant). **(b)** Analysis of texture features via extraction of nuclear patches of size 32 × 32 pixels with stride size of 16 pixels in either direction. **(c)** Table indicating differences in accuracy between patch-based classification and classification based on full nuclear images for both neural and linear models.

Particular known features for cancer progression are alterations in chromatin condensation patterns^42^. We measured chromatin condensation by plotting the heterochromatin (more condensed chromatin) to euchromatin (more open chromatin) ratio (HC/EC) as explained in Methods and in Supplementary Fig. 4. The threshold value for identifying heterochromatin was calculated using equation (2). The HC/EC ratio was found to be lower in normal BJ fibroblast cells and Human Mammary Epithelial Cells (HMECs) as compared to fibrocystic MCF10A cells and metastatic MCF7 cells (Fig. 3a). In agreement with previous studies there was a progressive increase in HC/EC ratio from normal to metastatic cell lines^12^.

The HC/EC ratio only provides a global view of chromatin condensation. Since the typical length scale of individual heterochromatin foci is in the range of a few hundred nanometers, we partitioned the nucleus into patches of 32 × 32 pixels (each pixel being 120 nm in width) as shown in Fig. 3b. These patches were then fed into our machine learning pipeline. The number of patches in each condition and the split into training and validation is given in Supplementary Fig. 5. As shown in Fig. 3c, both the neural model and the linear models (logistic regression, linear kernel support vector machine (SVM), and random forest classifier) did slightly worse on the patches (BJ versus MCF10A: 78.8% validation accuracy and 84.3% training accuracy for the neural model versus 70.8% validation accuracy and 73.0% training accuracy for the best linear model), since when analyzing patches the shape of the nucleus cannot be used for classification. This analysis confirms the discriminatory power of texture features at the length scale of heterochromatin foci for classification of normal versus cancer cell lines.

**Figure 4:**
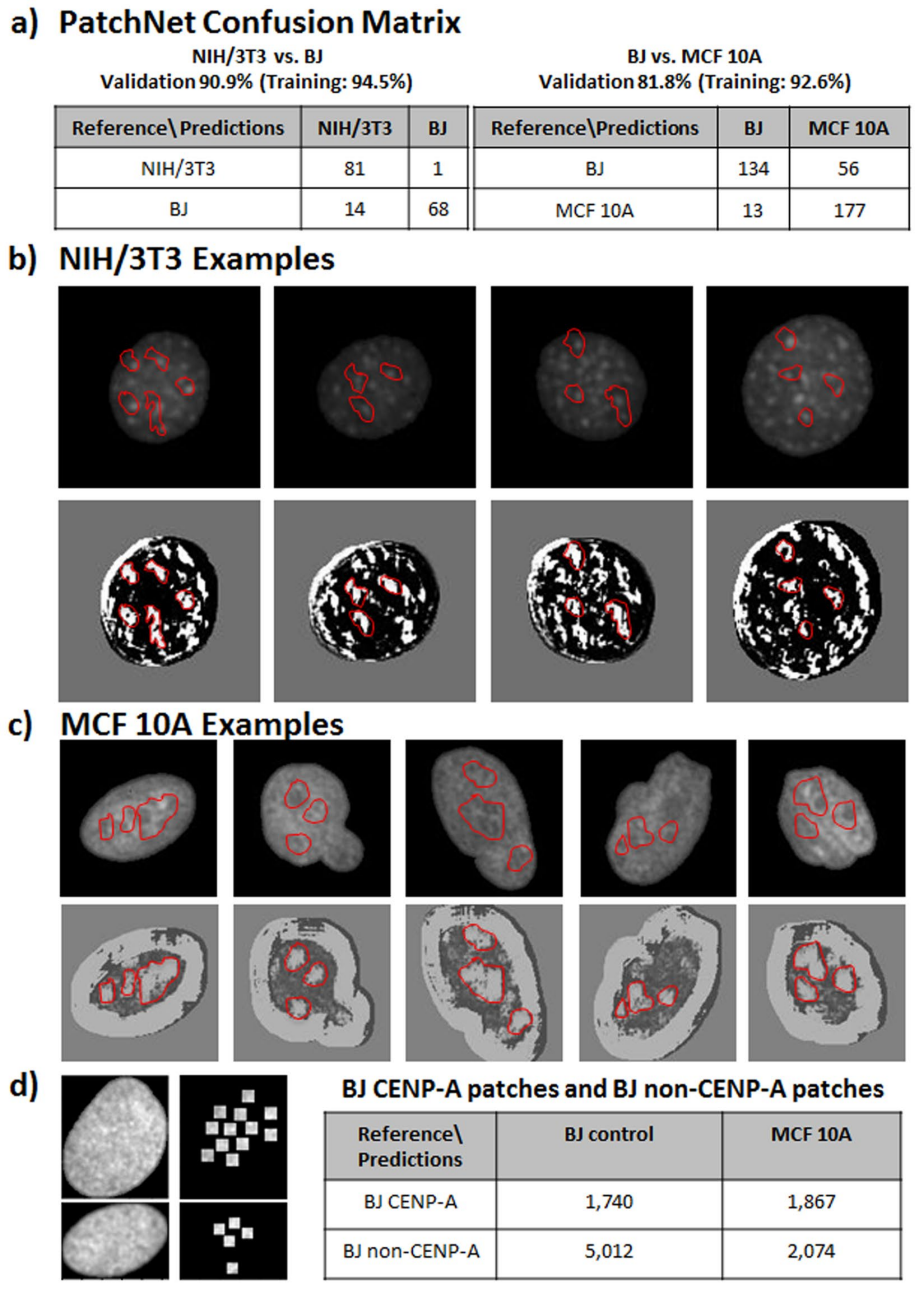
Functional annotation of the features identified based on a patch-analysis. **(a)** The confusion matrices for PatchNet in classifying between NIH/3T3 and BJ as well as between BJ and MCF10A. **(b)** Representative examples of NIH/3T3 nuclei (upper row) together with the features indicative of NIH/3T3 (shown in white) as learned by PatchNet (bottom row). The red masks show examples of regions that were identified by PatchNet as being indicative of NIH/3T3. The predicted NIH/3T3 indicative regions correspond to heterochromatin foci. **(c)** Representative examples of MCF10A nuclei (upper row) together with the features indicative of MCF10A (shown in white) as learned by PatchNet (bottom row). The red masks show examples of regions that were identified by PatchNet as being indicative of MCF10A. The predicted MCF10A indicative regions correspond to interfaces between heterochromatin and euchromatin regions. **(d)** BJ nuclei were stained with CENP-A and patches of size 11 × 11 pixels were extracted around the CENP-A marks (left image). The table compares the classification accuracy between CENP-A and non-CENP-A patches, showing that CENP-A patches are more often misclassified as MCF10A.

In general, tuning the patch size allows for trading off classification accuracy versus interpretability of the features. In order to better understand the features learned by our neural model when classifying between patches, we further reduced the patch size. To classify between NIH/3T3 and BJ nuclei we used patches of size 11 × 11 pixels and for classifying between BJ versus MCF10A nuclei we used patches of size 17 × 17 pixels to allow for more context in this more difficult classification task. The theoretical foundations for such an approach are described in PatchNet^43^. As suggested by the theory, Fig. 4a shows that the classification accuracies were slightly reduced when using PatchNet as compared to classification based on the full nucleus image (NIH/3T3 versus BJ: 90.9% validation accuracy and 94.5% training accuracy; BJ versus MCF10A: 81.8% validation accuracy and 92.6% training accuracy). A particular filter resulting from the PatchNet analysis is shown in Fig. 4b and c. It shows that significant features used to classify between NIH/3T3 and BJ nuclei as well as BJ and MCF10A nuclei are regions of high luminance contrast. Such regions in NIH/3T3 nuclei correspond to the heterochromatin regions, whereas in MCF10A nuclei PatchNet identified large interface regions between euchromatin and heterochromatin as important features for classification. A complete list of all filters is given in Supplementary Fig. 6a and Fig. 6b.

In previous studies, Centromere Protein A (CENP-A) has been established as a biomarker for peri-centeromeric heterochromatin regions^44^. In order to provide a functional annotation of the features learned by the convolutional neural net trained on BJ and MCF10A nuclei, we analyzed the classification of our model on BJ patches of size 11 × 11 pixels that contained CENP-A markers as well as BJ patches of size 11 × 11 pixels that did not contain CENP-A markers (see Fig. 4d). As seen in this figure, the model learned to correctly classify a significant portion of the patches not containing CENP-A markers as BJ, while it incorrectly classified the majority of patches containing CENP-A markers as MCF10A. Importantly, this shows that our platform identified heterochromatin regions as early cancer biomarkers. This is in accordance with domain expertise as CENP-A stains are indicative of regions of DNA damage^45,46^.

### SCENMED platform discriminates between normal and cancer cells in breast tissue slices with high accuracy

**Figure 5:**
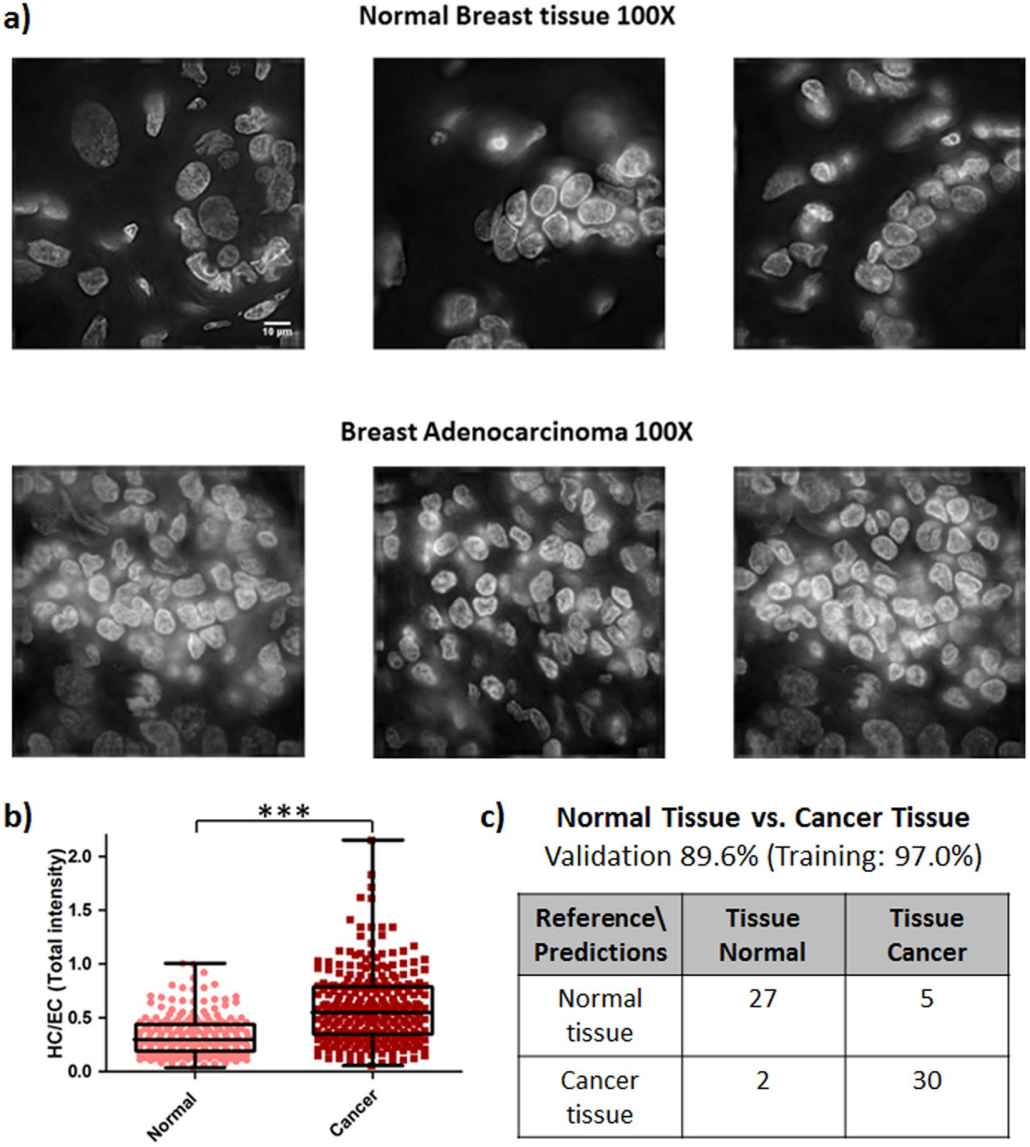
SCENMED analysis on tissue slices. **(a)** Representative DAPI-stained slices of normal and adenocarcinoma breast tissue (scale bar: 10 μm). **(b)** Whisker box plot of HC/EC ratio for normal (n = 217) and cancer tissue (n = 286); Student’s unpaired t-test ***p < 0.0001. **(c)** Confusion matrix for classification between nuclei cropped from cancerous and normal tissue slices.

We next validated our platform on normal and cancer tissue slices of human origin. Normal breast tissue slices used in our study consist of 10% glandular epithelium, 20% stroma and 70% adipose tissue, whereas the breast adenocarcinoma tissue slices consist of 100% cancer cells as per the pathology report provided in the datasheet. Representative wide-field images obtained using 100x magnification of both normal and cancer tissues stained with DAPI are shown in Fig. 5a; for representative images of the tissue sections in 20x magnification see Supplementary Fig. 7a. The nuclei were cropped from the DAPI stained images obtained using 100X objective as explained in Methods and Supplementary Fig. 7b. As shown in Fig. 5b, the nuclei from cancer tissue slices have a higher HC/EC ratio as compared to nuclei from normal tissue slices. We also validated the SCENMED pipeline on these nuclear images. As seen in the confusion matrices in Fig. 5c, SCENMED is able to accurately classify between nuclei from cancer and normal tissues (89.6% validation accuracy and 97.0% training accuracy).

### SCENMED’s sensitivity is benchmarked using nuclear mechanical perturbations that mimic cancer tissue microenvironments

**Figure 6:**
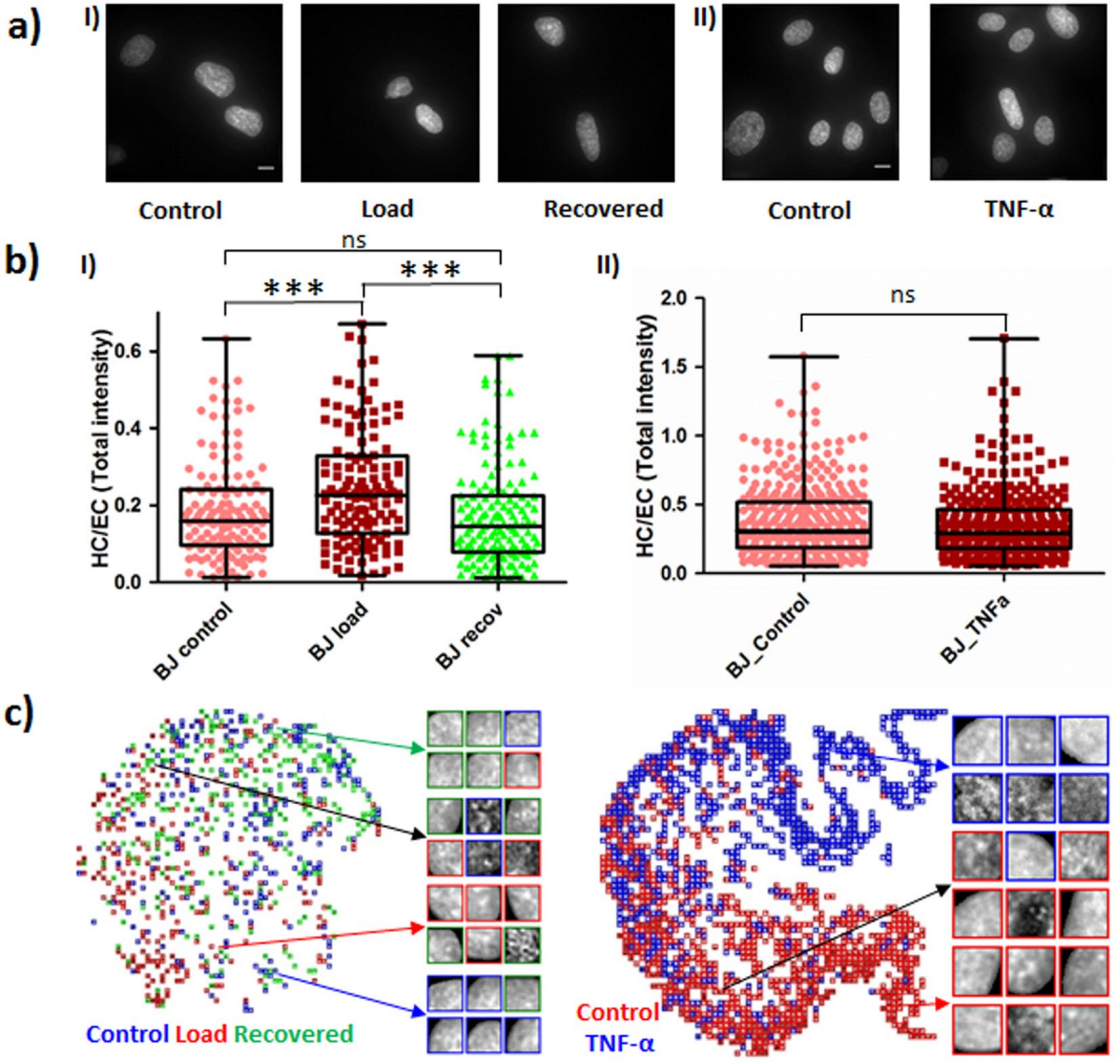
SCENMED analysis on cells subjected to nuclear mechanical perturbations. **(a)** Representative images of BJ nuclei under control, load and recovery conditions (in I) and under 30 minutes of TNF-α treatment (in II). **(b)** Whisker box plot for HC/EC ratio for BJ control (n = 134), load (n = 129) and recovery (n = 149) in (I) and control (n = 364) and TNF-α treated (n = 308) in (II); Student’s unpaired t-test ***p < 0.0001 and ns = not significant. **(c)** Corresponding t-SNE visualizations for BJ control, load, recovered nuclei patches as well as for BJ control and BJ TNF-α nuclei patches.

To test the sensitivity and benchmark our machine learning pipeline, the BJ cells were subjected to typical compressive forces as experienced by cells in the tissue microenvironment (in the order of micro-newtons of force per cell) for one hour and allowed to recover for one hour. The magnitude of compressive force applied was calculated using equation (1). In addition, we stimulated (for 30 min) BJ cells with tumor necrosis factor α (TNF-α), a cytokine that is known to play an important role in cancer progression and inflammation. The cells in each condition (Control, Load, Recovered, and after 30 min TNF-α stimulation) were fixed, permeabilized, and stained with DAPI (Fig. 6a). The resulting HC/EC ratios for these conditions are shown in Fig. 6b. Compressive load causes chromatin compaction as shown by the significantly elevated HC/EC ratio of load versus control cells. Interestingly, cells recovered their original HC/EC ratio after one hour of recovery, suggesting that normal cells possess an inherent structural memory in chromatin organization. Table 2, Fig. 6c and supplementary Fig. 8 show that the platform made more mistakes in distinguishing between recovered and control BJ nuclei than between load and control or recovered and control nuclei. This is especially apparent from the confusion matrix based on patches, since nearly half of the BJ control nuclei were wrongly classified as recovered nuclei, while the majority of load nuclei were classified correctly. The major reason for the indistinguishibility between BJ control and recovered nuclei is due to the highly reversible nature of the nuclear and chromatin patterns upon removal of load on these cells. While stimulation of BJ cells with TNF-α does not lead to a significant increase in the HC/EC ratio, our SCENMED platform was able to clearly distinguish between these two conditions. The classification accuracies and confusion matrices for these mechanical and chemical perturbations are shown in Table 2 and supplementary Fig. 8. In addition, the corresponding t-SNE plots are shown in Fig. 6c. These analyses indicate the sensitivity of our pipeline to detect even subtle changes in chromatin organization. Our mechanical and TNF-α stimulation assays can be seen as a model for disease progression, suggesting that our platform is applicable for early disease diagnostics.

**Table 2:** Confusion matrices for classification between BJ control, load, and recovered nuclei as well as confusion matrices for classification between BJ control and BJ TNF-α nuclei.

BJ control vs. BJ TNF-α							
Validation 86.1% (Training: 88.9%) (Full nuclei)			Validation 79.4% (Training: 76.8%) (Patches)				
Reference\Predictions	BJ control	BJ TNF-α	Reference\Predictions	BJ control	BJ TNF-α		
BJ control	125	23	BJ control	1,801	499		
BJ TNF-α	18	130	BJ TNF-α	445	1,855		
**BJ control vs. BJ load vs. BJ recovered**							
**Validation 55.8% (Training: 61.0%) (Full Nuclei)**				**Validation 45.4% (Training: 48.5%) (Patches)**			
**Reference\Predictions**	**BJ control**	**BJ load**	**BJ recovered**	**Reference\Predictions**	**BJ control**	**BJ load**	**BJ recovered**
BJ control	22	4	14	BJ control	90	68	148
BJ load	10	19	11	BJ load	50	173	83
BJ recovered	12	2	26	BJ recovered	65	59	182

## Discussion

A number of recent studies have shown that various diseases such as Cancer, Fibrosis, Alzheimer’s, and Premature Aging are accompanied by nuclear morphological alterations. These alterations often are a result of defective cytoskeletal-to-nuclear links as well as impaired mechanotransduction pathways^12,47–50^. The tissue microenvironment comprises of a large cellular heterogeneity in nuclear architecture. A growing number of evidence highlights that changes in the microenvironmental signals could potentially trigger a small group of cells into disease progression^18,51–54^. While detection of tissue-scale abnormalities have been easier to diagnose, early diagnostics of small groups of defective cells for example in tissue biopsies has been a major challenge. While challenging, early disease diagnostics has been identified as a major goal since it carries the promise of greatly increasing survival rates as well as reducing health care costs^55^.

Current approaches include genomic analyses to annotate mutations and alterations in the epigenome^56,57^. These methods are often based on population studies and hence their efficiency to detect abnormalities in single cells within tissue biopsies is low. The current approaches to diagnose abnormalities in tissue biopsies therefore include a combination of morphometric assays and immunohistochemistry, combined with genomic analyses. In order to reduce the subjectivity of the pathologist in classifying morphometric assays, recent efforts have introduced machine learning methods to this problem^30,31^. Such digital pathology approaches have included either using parametric or non-parametric approaches to detect alterations in tissue biopsies. While such efforts have proven to be beneficial in diagnosing late-stage diseases, early detection still poses a number of challenges. In addition, the deep learning approaches that often yield the highest accuracy, do not provide any interpretable or functional features that could aid the pathologist.

In this paper, we provided SCENMED, a single-cell nuclear mechano-morphometric diagnostic platform for cancer diagnosis. To benchmark our pipeline we subjected single cells to subtle microenvironmental alterations, thereby mimicking tumor microenvironments. Our platform robustly detected these subtle alterations that were reflected in chromatin condensation states. We also provided a quantitative analysis of our platform to discriminate normal and cancer cell lines as well as different kinds of cancer cell lines and cells in tissue biopsies. In addition to achieving high classification accuracy, our platform also provides interpretable features by performing the analysis on small regions within the cell nucleus. The nuclear mechano-morphometric features that we identified indicate typical length scales of chromatin condensation that are potentially important for early cancer diagnosis. Collectively, our platform has applications for early disease diagnosis in fine-needle biopsies and blood smears. Low-cost and fully automated methods based on nuclear mechano-morphometric biomarkers such as the one proposed here could also be combined with single-cell genomic methods and are attractive not only for clinical applications such as cancer diagnosis, but also open new avenues for drug discovery programs as well as the development of therapeutic interventions at single-cell resolution.

## Methods

### Cell culture, tissue slice preparation, immunofluorescence staining, and imaging

All experiments were performed in accordance with relevant guidelines and regulations at National University of Singapore (NUS). NIH/3T3 (CRL-1658), BJ (CRL-2522), MCF10A (CRL-10317), MCF7 (HTB22) and MDA-MB-231 (HTB-26) cells were obtained from ATCC. They were cultured in DMEM (ThermoFisher Scientific 11885092) media supplemented with 10% Fetal Bovine Serum (FBS) (ThermoFisher Scientific 16000044) and 1% pen-strep (Sigma P4333) antibiotic. Antibodies used: CENP-A (Cell Signalling Technology 2048 S), Histone H2A.X Phospho S139 (Abcam 184520). Human breast tissue sections within normal limits (CS708873, OriGene) and metastatic breast adenocarcinoma tissue sections (CS548359, OriGene) were purchased from OriGene Technologies where these samples were obtained according to the relevant ethical consenting practices (for more information visit http://www.origene.com/Tissue). Other reagents: Histozyme (H3292-15ML, Sigma) and ProLong® Gold Antifade Mountant with DAPI (P36941, ThermoFischer Scientific).

Cells were fixed with 4% parafoldehyde (PFA) (Sigma, 252549-500 ml) for 10 minutes. For compressive force experiments, cells were fixed in the presence of load, where the load and coverslip were removed after the fixation step. Cells were then permeabilized with 0.5% triton (Sigma, ×100-100 ml) for 10 minutes and rinsed with PBS after each step. Cells were then blocked with 5% BSA for 30 minutes and incubated with primary antibody followed by the secondary antibody as per the manufacturer’s instructions. Cells were then labeled with DAPI solution (ThermoFisher Scientific R37606) in PBS as recommended by the supplier. For experiments that did not involve antibody labeling, DAPI solution was added after the permeabilization step.

Formalin-fixed, paraffin-embedded (FFPE) tissue sections (5 μm thickness) on slides were deparaffinized by heating them in an oven at 60 °C for 5 minutes and subsequently washing them with xylene. The sections were then rehydrated in serially diluted ethanol solutions (100% − 50%) as per standard protocols and rinsed with water. Antigen retrieval was performed using Histozyme solution as per manufacturer’s protocol and then rinsed with water. DAPI was then added onto these sections and covered with a coverslip. The slides were incubated for 24 hours after which the coverslips were sealed and taken for imaging.

Images were obtained using an Applied Precision DeltaVision Core microscope. Wide field images were obtained using 60X objective (air, NA 0.7) with a pixel size of 0.2150 µm. These 512 × 512 12-bit images were deconvolved (enhanced ratio, 10 cycles) and saved in tiff format. The images and their corresponding labels (e.g. NIH/3T3, NIH/3T3_Load) were then stored in HDF5 format for subsequent analysis.

### Compressive loading assay

1000 cells were seeded on glass bottom dishes (Ibidi 81158) and allowed to attach overnight. Then a pluronic acid treated coverslip was placed on the cells. The compressive force was applied by placing parafilm-wrapped metallic nuts (mass = 22 g) on the coverslip. This corresponds to each cell experiencing a force in the order of micro-newtons (calculation shown below). For recovery, the metallic nut was removed and the cells were allowed to recover for one hour before being fixed for immunofluorescence staining.

The force was computed as follows:

Mass = 22 g (22 × 10^−3^ kg); force applied (weight) = mass × acceleration due to gravity = 22 × 10^−3^ × 10 = 220 mN;

1 $$\frac{force}{cell}=\frac{compressive\,force-buoyant\,force\,due\,to\,1ml\,media}{number\,of\,cells}$$

Buoyant force = weight of volume of media displaced; volume of media displaced by a single nut = 1 ml (1 g); mass equivalent to 1/20^th^ of the nut that is submerged in the media during the experiment = 1.1 g; number of cells = 1000.

$$\frac{force}{cell}=\frac{(22-1.1)\times {10}^{-3}\times 10}{1000}=209\,{\boldsymbol{\mu }}{\boldsymbol{N}}$$

### Image Processing and Segmentation

Our goal in segmentation was to automatically segment cleanly imaged nuclei from potentially noisy microscope images. Hence we focussed on precision in our segmentation methods.

We identified three types of noise introduced by the microscope imaging procedure. We refer to these types of noise as overexposure, edge blur, and drift blur. We define a nucleus to be overexposed when at least 25% of the nucleus has a pixel value higher than 3700. We define edge blur as the noise introduced by de-convolution at nuclei along the edge of the 512 × 512 image frame. We define drift blur to be the noise introduced by nucleus motion during the imaging process. We filtered out overexposed nuclei and removed edge blurred nuclei with software methods as described in the following paragraph, while drift blurred image sets were identified and removed by testing if a given image set for a particular nucleus line was distinguishable from the other image sets for the same nucleus line using a neural net approach (see implementation section for more details).

We compared a variety of segmentation algorithms (Supplementary Fig. 2) and found that the following procedure provided the best results for identifying the nuclei, namely marker-based watershed segmentation to segment the nuclei followed by software filtering techniques to remove nuclei that are affected by noise. To select appropriate markers for watershed, we first used morphological reconstruction, which resulted in better nucleus separation. Then, we used a Sobel edge detector to identify nucleus edges, noting that this detector also identified intra-nucleus edges. Next, we marked edges identified by the edge detector as foreground, and correspondingly marked pixels with values within the lowest 1% of image pixel values as background. Finally, we ran marker-based watershed segmentation to identify the nuclei.

After segmentation, we removed any nuclei that were larger than 128 × 128 pixels as well as any nuclei that were overexposed. For nuclei along the edges of an image, we cropped a depth of 15 pixels along the blurred edge. Nuclei patches were extracted from 128 × 128 crops by using a 32 × 32 sliding window approach with a stride of 16 in either direction. We only used patches that consisted of at least 85% nucleus. We also removed overexposed patches using the same technique that we used to remove overexposed nuclei.

For tissue slices, the image analysis was performed using FijiImageJ. The schematic is shown in Supplementary Fig. 7b. The nuclei in the original image were first selected using auto-threshold “Shanbag dark.” Nuclei were separated by applying a Gaussian blur to the selected regions, converting them into a binary image, and finally applying watershed. Each identified nucleus was then saved as a separate image for further analysis.

### HC: EC calculation

First, the nucleus was identified by the Otsu thresholding method (Supplementary Fig. 4). To measure chromatin condensation in each nucleus a custom macro was written in FijiImageJ.

The threshold for identifying heterochromatin was calculated as:

2 $$min({\rm{p}}{\rm{i}}{\rm{x}}{\rm{e}}{\rm{l}}\,{\rm{i}}{\rm{n}}{\rm{t}}{\rm{e}}{\rm{n}}{\rm{s}}{\rm{i}}{\rm{t}}{\rm{y}})+0.6\,[max\,({\rm{p}}{\rm{i}}{\rm{x}}{\rm{e}}{\rm{l}}\,{\rm{i}}{\rm{n}}{\rm{t}}{\rm{e}}{\rm{n}}{\rm{s}}{\rm{i}}{\rm{t}}{\rm{y}})\,-\,min\,({\rm{p}}{\rm{i}}{\rm{x}}{\rm{e}}{\rm{l}}\,{\rm{i}}{\rm{n}}{\rm{t}}{\rm{e}}{\rm{n}}{\rm{s}}{\rm{i}}{\rm{t}}{\rm{y}})]$$

The HC/EC value is then calculated where HC is the total intensity (heterochromatin regions) and EC is the difference between the total intensity of the entire nucleus and the total intensity of the heterochromatin regions.

### Deep Learning

Inspired by the recent success of deep learning in image classification^58,59^, we modified state-of-the-art convolutional neural networks (CNNs) to perform nucleus classification on both, full nucleus images and nucleus patches. CNNs are particularly suited for this problem, since the convolution operation provides translational invariance and hence the output is invariant with respect to the position of a feature within a nucleus. In particular, we implemented an adaptation of the Visual Geometry Group (VGG) architecture with state-of-the-art layers, activations, and initializations^60^.

For our CNN, we first used batch normalization (with scaling) to normalize input images. Then we used 10 convolutional layers interspersed with max pooling following the conventions in VGG. Namely, we only used convolutions with kernel size 3 and began convolutions with 32 filters, doubling the number of filters each time after using a max pooling operation. We used two fully connected layers with dropout connections after the convolution layers. Each of our convolution layers has Parametric Rectified Linear Unit (PReLU) activations, the fully connected layers have Rectified Linear Unit (ReLU) activations, and the output layer has a softmax activation.

We used a weighted cross entropy loss and trained our network using the Adam optimizer, a variant of stochastic gradient descent^61^. We set class weights proportional to the amount of each class in the training set in order to maintain higher precision and recall. We initialized the learning rate to 8e-5. We used batch sizes of 32 for full nucleus images and batch sizes of 128 for nucleus patches. We initialized all weights according to the variant of the Xavier initialization proposed in He *et al*.^58^ and used the patience method described in Goodfellow *et al*.^60^ with a patience limit of 20 steps to enforce early stopping, which prevents overfitting.

### PatchNet

In order to visualize and interpret features learned by convolutional neural networks, we used PatchNet^43^. PatchNet is composed of a small classifier CNN that is applied across patches of the original image. The classification decisions across all patches are then averaged in order to predict the class of the entire input image. PatchNet provides feature interpretability by providing a global heat-map (based on classification decisions from each patch) as well as filter heat-maps (based on filter activations from the last convolutional layer). By hand-selecting patch sizes for each of our classification problems, we were able to extract interpretable heat-maps from PatchNet without sacrificing accuracy.

In classifying between NIH/3T3 and BJ nuclei, we used patches of size 11 × 11 since the model was able to achieve a high accuracy by simply using areas of high luminance contrast to identify NIH/3T3 nuclei. However, in classifying between BJ nuclei and MCF10A nuclei, we used larger patches of size 17 × 17 to allow the network to incorporate more local context to improve classification at the expense of heatmap granularity.

### Linear Models and Feature Selection

In order to better understand the effects of different features on learning, we also trained three linear models (logistic regression, linear SVM, and random forest classifier) on hand-selected features (as described below).

We studied the impact of both, shape-based features and texture-based features, for nucleus classification by using the following feature set (containing 104 features for full nucleus images and 99 features for nucleus patches). For shape-based features, we used area, eccentricity, major length axis, minor length axis, and roundness as features. For texture-based features, we used local binary patterns with a radius of up to 3 and 8-bit point codes (256 possible codes), parameter free Threshold Adjacency Statistics (PFTAS), and Zernike moments. We also considered gray level run length and co-occurrence matrices as well Gabor wavelet features. But since extracting these features is highly computationally intensive and did not improve accuracy, we did not include these features in the analysis.

We trained a weighted L2-Regularized Logistic Regression with the L-BFGS solver on our datasets. For BJ vs. MCF 10 A nucleus classification, we also trained a linear SVM as well as a random forest classifier. The linear SVM was trained using the default parameters in the python sci-kit learn library. The random forest classifier was trained using 100 estimators and a maximum depth of 8. To search across the parameter space for random forest model selection, we used a randomized search across a subset of possible parameters. Kernel SVMs (such as radial basis SVMs and kernels based on optimal transport) were too computationally expensive to train on the nucleus patches datasets, but also tended to over-fit on full nucleus datasets. Hence, we did not include these models in the results.

### Evaluation of model performance

We used a held-out validation set to determine model generalizability. We selected our validation set using a fair split of examples among the classes to total at most 15% of the entire data set. Performance of the neural model was determined based on the accuracy on the validation set. We chose not to use area under the receiving operating characteristic curve or precision and recall as metrics, but instead to explicitly list out the confusion matrices. Performance of the linear model was determined solely by accuracy on the validation set. More importantly, we used feature ablation tables for the linear model to analyze the significance of each feature in classification.

### Implementation

A key challenge in automatically segmenting cleanly imaged nuclei was to account for drift blur. The main challenge with drift blur is that the texture is destroyed, but the pixel value histograms for such images are indistinguishable from those of cleanly imaged nuclei. Thus, to identify drift blur in an image set, we applied our neural models to attempt to distinguish between nuclei that were segmented from different image sets (taken on different days), but are from the same cell line. We used a pairwise comparison across all image sets from the same cell line, and if the model was able to distinguish between one image set and all the others with a validation accuracy of consistently greater than 60%, we discarded such datasets. We used 60% as our threshold, even though we would ideally prefer complete indistinguishability (corresponding to a threshold of 50%), since some variability between the different image sets is to be expected. In addition, we manually verified that the files that were discarded contained a majority of nuclei with drift blur.

We now describe the relevant technologies used in our dataset and model development. Our image processing and segmentation pipeline was implemented in python using the libraries scikit-image^62^ and scipy^63^. Our neural models were implemented in python using Tensorflow and trained on an NVIDIA Titan X Pascal 12GB GPU. Our logistic regression model was implemented in python using scikit-learn and feature extraction for the linear model was done using the mahotas python library. Image segmentation on an NVIDIA Titan X Pascal 12GB GPU took roughly 0.5 seconds per 512 × 512 image containing several nuclei. As an example, training our CNN for classifying BJ vs. NIH/3T3 nuclei based on the full 128 × 128 nucleus images took less than 900 seconds on an NVIDIA Titan X Pascal 12GB GPU. More precisely, the model required 56 epochs until convergence, where each epoch consisted of 20 mini-batches composed of 128 images split equally among the two classes, and a complete forward and backward pass across all 20 mini-batches in an epoch took 14.66 seconds.

### Data availability

Data and code for segmentation and neural network training will be made available on github upon publication of this paper.

## Electronic supplementary material

Supplementary information
