# Supplementary material for: Machine Learning for Nuclear Mechano-Morphometric Biomarkers in Cancer Diagnosis
Source: Sci Rep. 2017 Dec 20;7:17946. doi: 10.1038/s41598-017-17858-1 (PMC5738417; doi:10.1038/s41598-017-17858-1)
Supplement: Supplementary file 1 — Supplementary information [file 41598_2017_17858_MOESM1_ESM.pdf]

Machine Learning for Nuclear Mechano-Morphometric Biomarkers in Cancer Diagnosis

Adityanarayanan Radhakrishnan*, Karthik Damodaran*, Ali C. Soylemezoglu, Caroline Uhler and GV Shivashankar

* equal contribution; corresponding authors: shiva.gvs@gmail.com, cuhler@mit.edu;

Supplementary Figure Captions:

Supplementary Figure 1: Image processing. **(a)** Representative images showing three different kinds of noise that was detected in the microscope images: (1) overexposure, (2) edge blur due to deconvolution along the edges of the image, (3) blur due to drift. **(b)** Nucleus segmentation is performed using the following steps: (1) reconstruction by dilation, (2) Sobel Edge detection, (3) Marker Selection, (4) Marker-Controlled Watershed Segmentation.

Supplementary Figure 2: A comparison of different nucleus segmentation methods. The top row contains a representative sample of original images before segmentation. The second row contains the segmented images after applying the Otsu's thresholding algorithm. The third row shows the resulting segmented images after applying raw marker-based watershed. The fourth row shows the results obtained by applying our proposed segmentation algorithm by combining a marker-based watershed approach with morphological operations on the original image. The last row shows the resulting crops after application of our segmentation method.

Supplementary Figure 3: Classification of NIH/3T3 versus BJ, BJ versus MCF10A, and MCF10A versus MCF7 versus MDA-MB-231 based on whole nuclei images. **(a)** Table summarizing the amount of training and validation data for the 3 classification tasks as well as the split in the amount of data for each class. **(b)** Plots of training and validation losses for each of the three classification tasks.

Supplementary Figure 4: Schematic of custom macro for measuring HC:EC ratio from nucleus crops.

Supplementary Figure 5: Table summarizing the amount of training and validation data for full nuclei and patches used for classification as well as the split of data among classes.

Supplementary Figure 6: Global heat-maps from PatchNet, a visualization of all filters from PatchNet, and confusion matrices/validation accuracies for PatchNet models. **(a)** Global heat-maps for representative NIH/3T3 and BJ nuclei, a comparison of filter activations across all 64 filters for the selected samples, and confusion matrix/validation accuracy for NIH/3T3 and BJ nuclei for the PatchNet model. **(b)** Global heat-maps for representativie BJ and MCF10A nuclei, a comparison of filter activations across all 64 filters for the selected samples, and confusion matrix/validation accuracy for BJ and MCF10A nuclei for the PatchNet model.

Supplementary Figure 7: Tissue section nuclei images and their segmentation **(a)** Low magnification (20X objective) wide field images of normal and adenocarcinoma breast tissue

sections stained with DAPI. **(b)** Schematic for generation of nucleus crops from wide field images (100X objective) of DAPI stained tissue sections.

Supplementary Figure 8: Table summarizing the amount of training and validation data for classifying between BJ control, load, and recovered nuclei patches as well as for classifying between BJ control and BJ TNF-α nuclei patches.

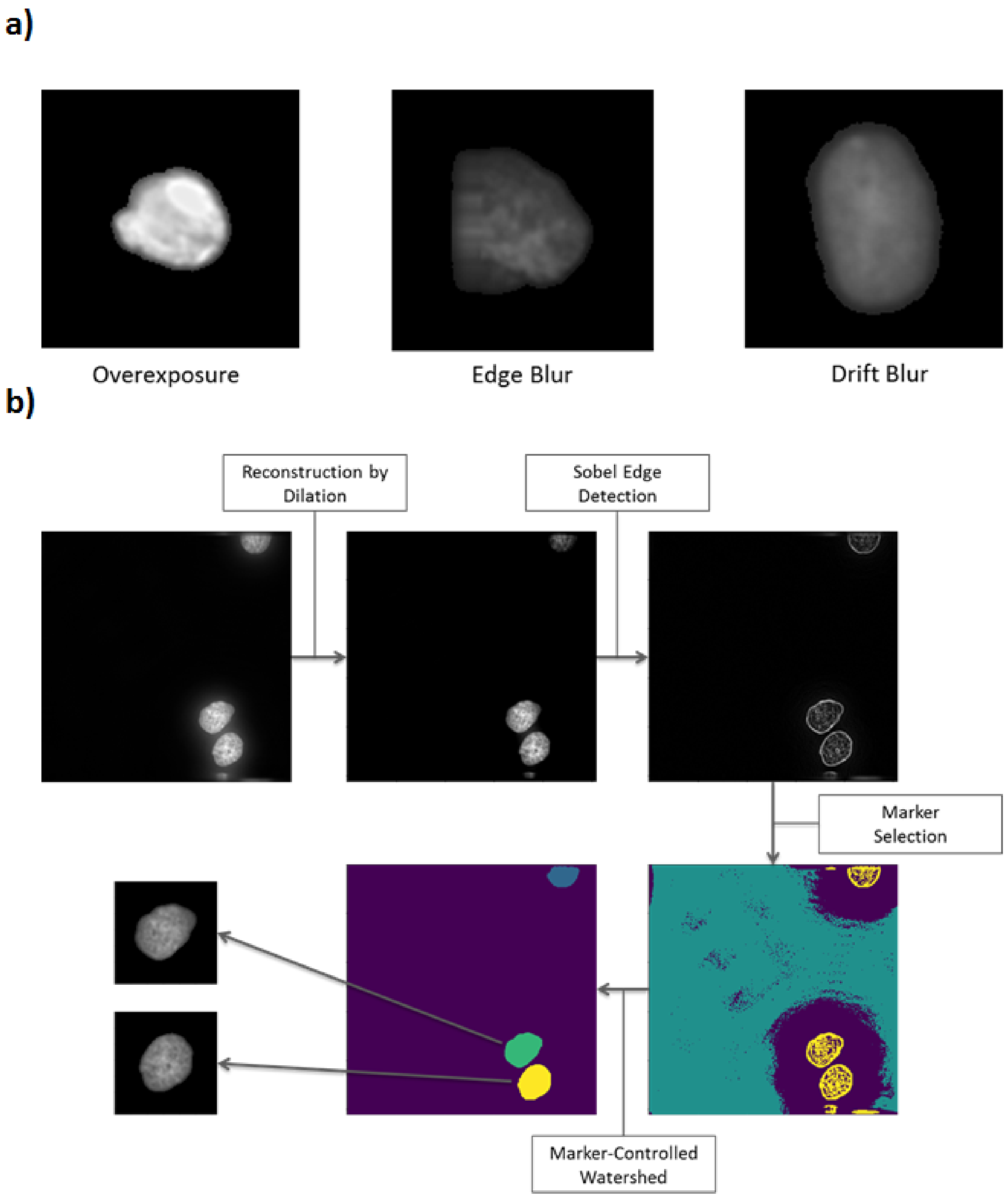

Supplementary figure 1

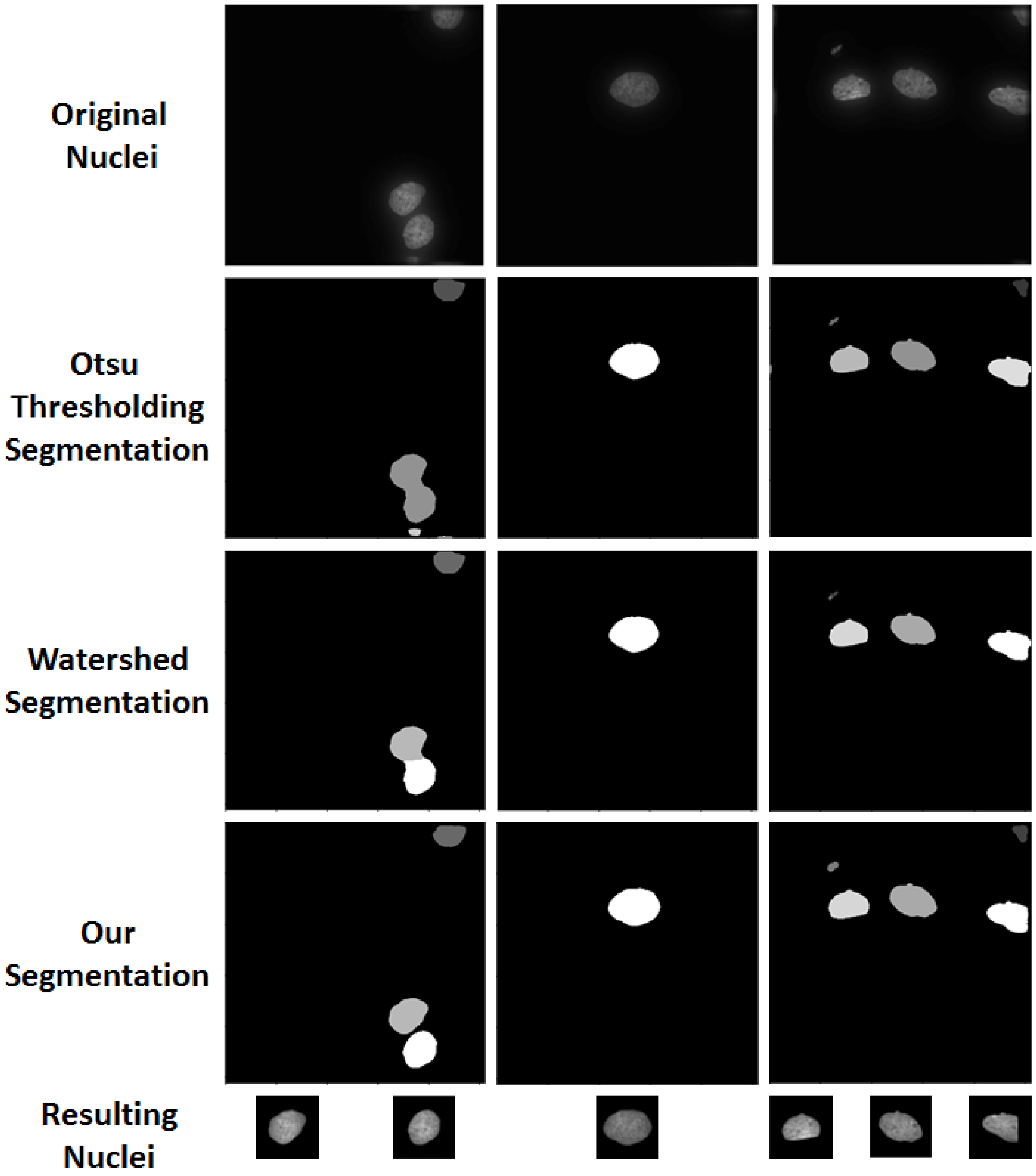

Supplementary figure 2

a)

Cell Types	Amount of Training Data	Amount of Validation Data	Split Among Classes
BJ vs. NIH/3T3	5285 nuclei	932 nuclei	2067 BJ, 4150 NIH/3T3
BJ vs. MCF 10A	2484 nuclei	380 nuclei	1267 BJ, 1597 MCF 10A
MCF 10A vs. MCF7 vs. MDA-MB-231	3253 nuclei	573 nuclei	1196 MCF7, 640 MCF 10A, 1990 MDA-MB-231

b)

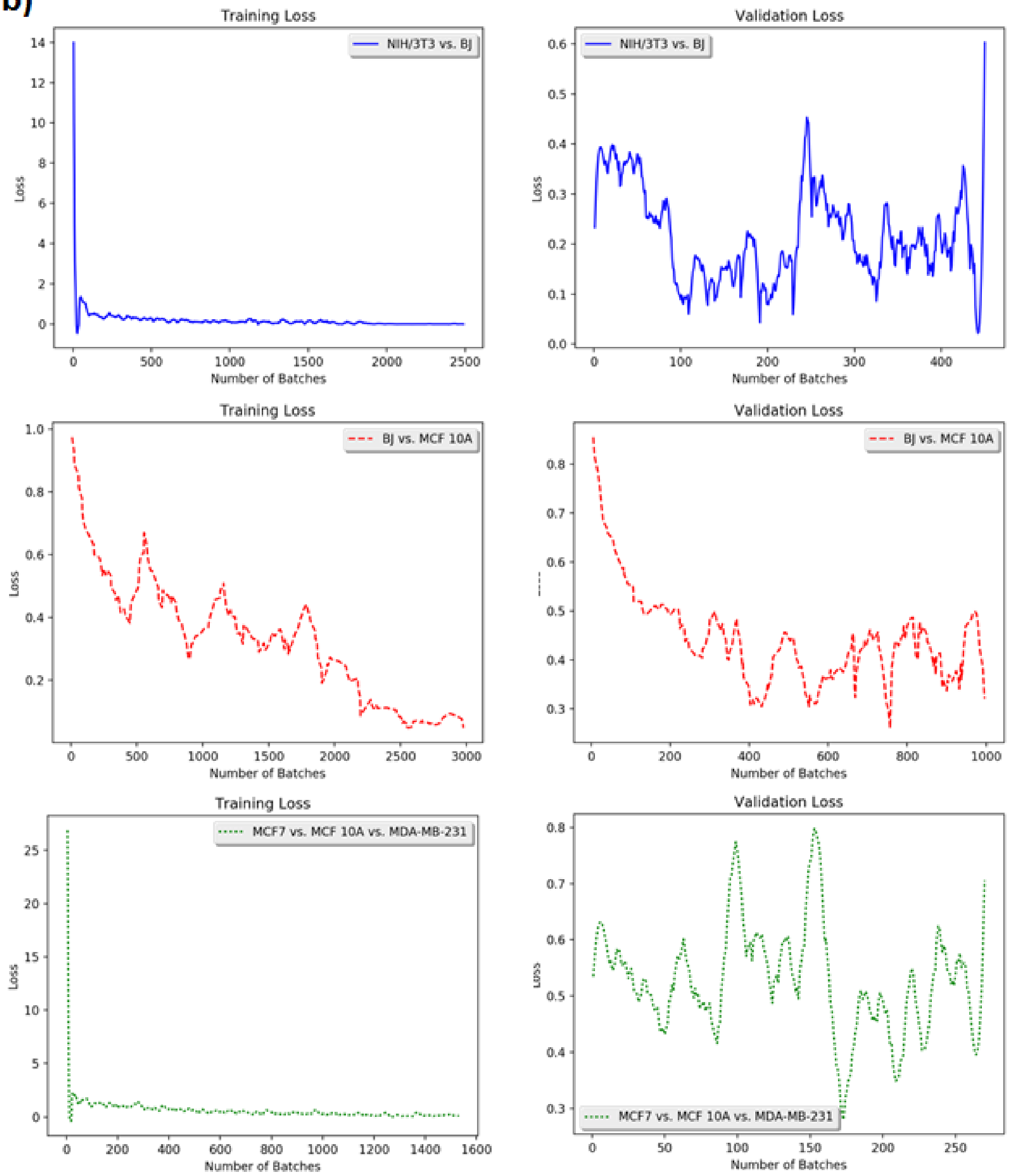

Supplementary figure 3

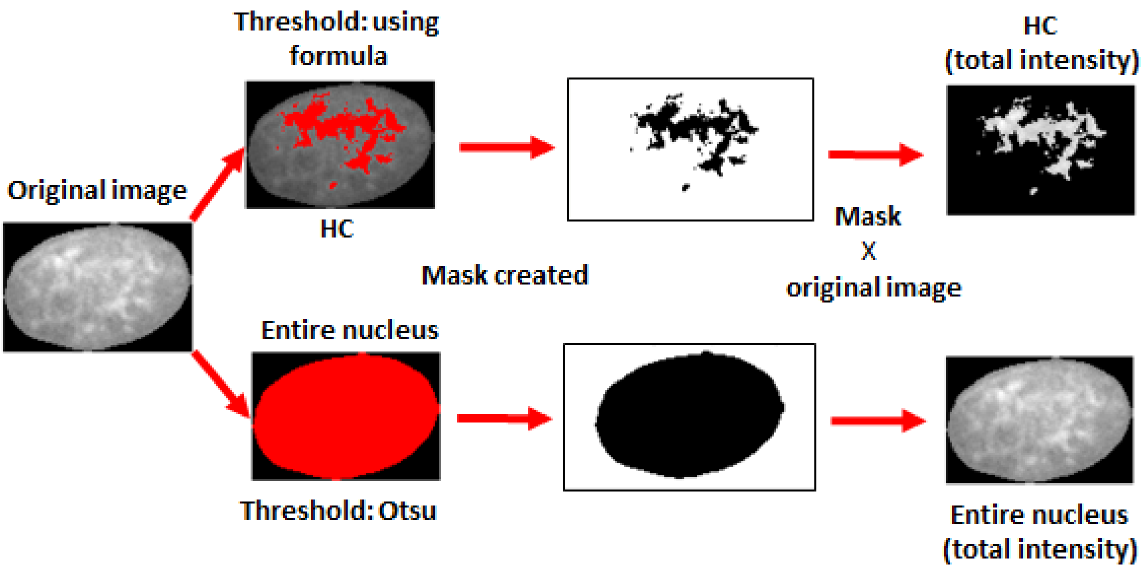

Supplementary figure 4

BJ vs. MCF 10A

Data Type	Amount of Training Data	Amount of Validation Data	Skew Among Classes
Full Nuclei	2484	380	1597 MCF 10A, 1267 BJ
Patches	29755	4600	19014 MCF 10A, 15341 BJ

Supplementary figure 5

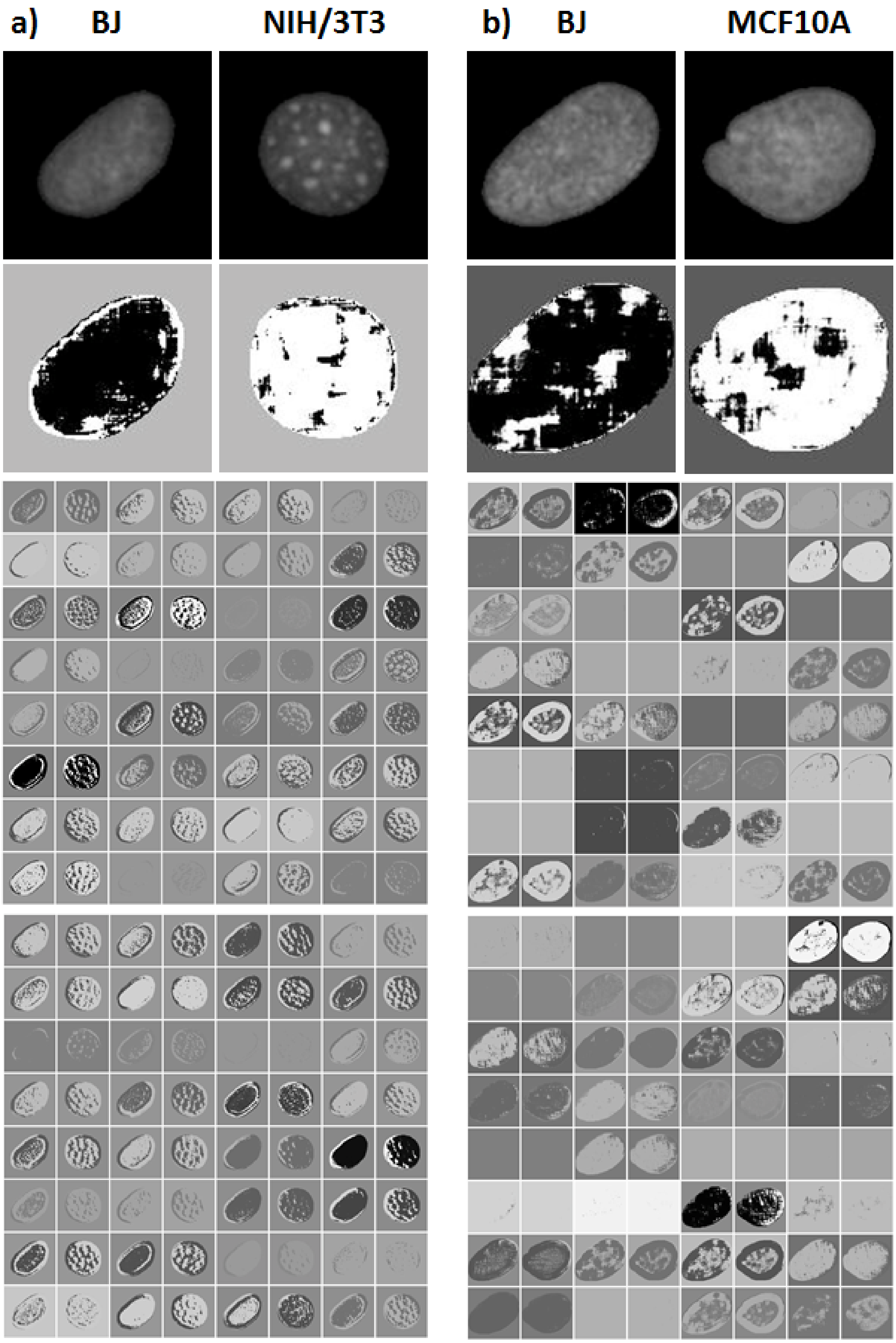

Supplementary figure 6

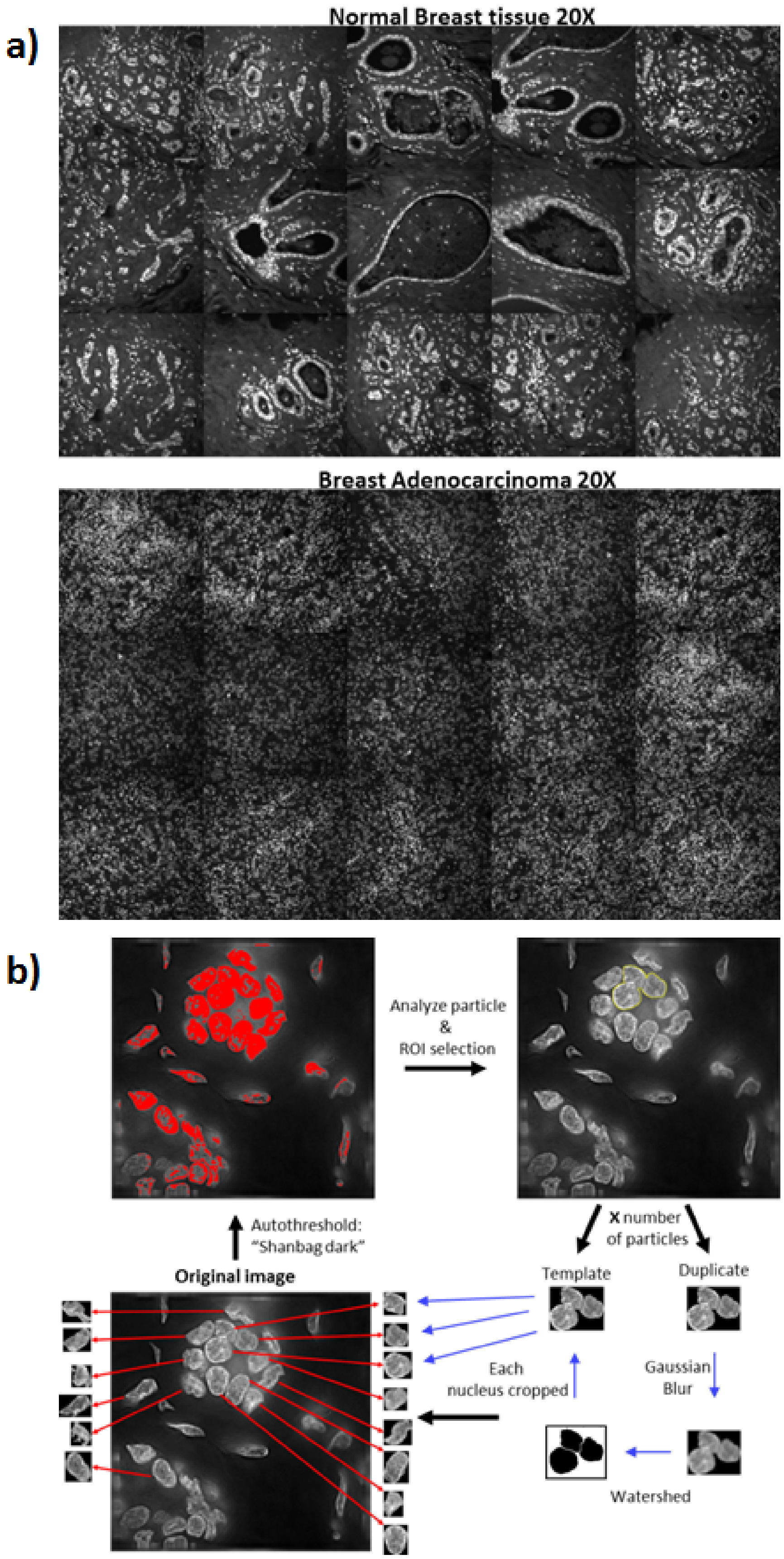

Supplementary figure 7

Cell Types	Amount of Training Data	Amount of Validation Data	Split Among Classes
**BJ control vs. load vs. recovered**	6786 nuclei	918 nuclei	2996 BJ control, 2040 BJ load, 2668 BJ recovered
**BJ control vs. BJ TNF-α**	26690 nuclei	4600 nuclei	15341 BJ, 15949 TNF-α

Supplementary figure 8
